# Multi-Targeting Intranasal Nanoformulation as a Therapeutic for Alzheimer’s Disease

**DOI:** 10.3390/biom13020232

**Published:** 2023-01-25

**Authors:** Oksana Fihurka, Yanhong Wang, Yuzhu Hong, Xiaoyang Lin, Ning Shen, Haiqiang Yang, Breanna Brown, Marcus Mommer, Tarek Zieneldien, Yitong Li, Janice Kim, Minghua Li, Jianfeng Cai, Qingyu Zhou, Chuanhai Cao

**Affiliations:** 1 Department of Pharmaceutical Sciences, Taneja College of Pharmacy, University of South Florida, Tampa, FL 33612, USA; 2 Department of Neurology, College of Medicine, University of South Florida, Tampa, FL 33612, USA; 3 Department of Chemistry, College of Arts & Sciences, University of South Florida, Tampa, FL 33612, USA; 4 USF-health Byrd Alzheimer Institute, Tampa, FL 33612, USA

**Keywords:** melatonin, insulin, THC, intranasal treatment, APP/PS1 mice, Alzheimer’s disease

## Abstract

Melatonin, insulin, and Δ9-tetrahydrocannabinol (THC) have been shown to reverse cognitive deficits and attenuate neuropathologies in transgenic mouse models of Alzheimer’s disease (AD) when used individually. Here, we evaluated the therapeutic properties of long-term intranasal treatment with a novel nanoformulation containing melatonin, insulin, and THC in aged APPswe/PS1ΔE9 (APP/PS1) mice, a transgenic model of AD. Transgenic mice at the age of 12 months were intranasally administered with a new nanoformulation containing melatonin, insulin, and THC at doses of 0.04, 0.008, and 0.02 mg/kg, respectively, once daily for 3 months. The spatial memory of the mice was assessed using the radial arm water maze (RAWM) test before and after drug treatment. Brain tissues were collected at the end of the treatment period for the assessment of Aβ load, tauopathy state, and markers of mitochondrial function. The RAWM test revealed that the treatment with the melatonin–insulin–THC (MIT) nasal spray improved the spatial learning memory of APP/PS1 mice significantly. Results of protein analyses of brain homogenates indicated that MIT treatment significantly decreased the tau phosphorylation implicated in tau toxicity (*p* < 0.05) and the expression of CKMT1 associated with mitochondrial dysfunction. Moreover, MIT significantly decreased the expression of two mitochondrial fusion-related proteins, Mfn2 and Opa1 (*p* < 0.01 for both), while increasing the expression of a mitophagy regulator, Parkin, suggesting a compensatory enhancement of mitophagy due to MIT-promoted mitochondrial fusion. In conclusion, this study was the first to demonstrate the ability of an MIT nanoformulation to improve spatial memory in AD mice through its multi-targeting effects on Aβ production, tau phosphorylation, and mitochondrial dynamics. Thus, MIT may be a safe and effective therapeutic for AD.

## 1. Introduction

Alzheimer’s disease (AD) is a debilitating condition that impairs memory, thought processes, and cognitive function primarily in older adults. The pathological features of AD include amyloid-β (Aβ) deposits in senile plaques, intracellular neurofibrillary tangles composed of hyperphosphorylated tau (p-tau), and synaptic dysfunction accompanied by neuroinflammation and mitochondrial and metabolic dysfunctions [1,2,3,4,5]. In this regard, AD is a complex and multifaceted ailment that requires a multifaceted therapeutic approach. Though AD is a global medical research priority with the rapidly aging society worldwide, little progress has been made in the development of effective treatments for AD [6]. Although several drugs have been approved by the US Food and Drug Administration (FDA) for the treatment of AD, they improve the behavioral symptoms of AD only temporarily with moderate efficacy [7]. Therefore, there is an unmet need for more efficacious therapies that can prevent, delay, or halt the pathogenesis and progression of AD by intervening in multiple prominent pathological processes leading to neuronal loss and cognitive decline. Since multiple factors have been linked to the onset of AD, it is believed that a multi-target therapy consisting of a combination of medications will be the most effective therapeutic approach for AD, and an effective combinational AD treatment should have a synergistic effect targeting the broad range of pathological factors of the disease [8].

Recently, Shukla et al. revealed the mechanism by which melatonin inhibited the amyloidogenic processes of β-amyloid aggregate formation and promoted cell pro-survival pathways [9]. In the last several years, hundreds of publications have confirmed that melatonin, acting as an endogenous broad-spectrum free radical scavenger and antioxidant, is highly beneficial for mitochondrial function support [10,11]. As the production of melatonin decreases with aging, a significant decrease in the melatonin excretion capacity has been found in patients with AD compared with the age-matched control subjects [12]. In fact, aging itself presents a unique challenge due to inherent mitochondrial dysfunction and the prevalence of chronic metabolic diseases. Incidentally, metabolic dysfunction is a well-established feature of AD, evidenced by brain glucose hypometabolism that can be observed potentially decades prior to the development of AD symptoms [5,13]. The reduced insulin levels and activity contribute to the pathological processes that characterize AD, while restoring insulin to normal levels in the brain provides therapeutic benefits to adults with AD [14]. Though insulin is a good candidate to fight AD, bioavailability becomes a major issue since the blood–brain barrier (BBB) blocks drug entry into the brain to exert its desired pharmacologic effect. The intranasal insulin treatment developed by William H. Frey for AD has been shown to improve memory in both Alzheimer’s patients and normal adults in multiple clinical trials [15,16,17,18]. A non-invasive intranasal method allows for the bypassing of the BBB and the rapid delivery of therapeutic agents to the brain along the olfactory and trigeminal neural pathways [19,20]. Intranasal administration avoids pre-systemic elimination, reduces systemic exposure, and minimizes unwanted side effects, which is beneficial for low-dose therapy with molecules such as THC. Mounting evidence has indicated that THC and other compounds found in marijuana can promote the intraneuronal removal of the toxic protein Aβ [21,22]. According to researchers at Tel Aviv University, treatment with THC at a low dose triggered an endogenous compensatory mechanism that improved cognitive function in aged mice [23]. In line with their findings, our previous studies have discovered that continuous low-dose THC intranasal treatment exhibited multiple effects on AD-related pathological changes, including the inhibition of Aβ production and aggregation, a reduction in tau hyperphosphorylation and enhancement of mitochondrial function [24,25].

Recent advances in drug formulation design and development have allowed us to develop a nanoformulation to improve drug delivery into the brain [26,27,28,29,30]. Building upon the background above and our research achievements, we tested the effect of intranasal treatment with a new nanoformulation containing melatonin, insulin, and THC on AD-related cognitive impairment and pathologic changes using an AD mouse model. The MIT nasal formula was developed to cover multiple drivers of AD pathogenesis. Since all the components of the MIT nanoformulation are either nutraceuticals or FDA-approved drugs, this nanoformulation is safe for long-term application. Since the mechanism of each component is well known, the MIT nanoformulation is expected to hit multiple targets involved in AD pathogenesis and progression.

## 2. Materials and Methods

### 2.1. Animals and Materials

Double transgenic APP_swe_/PS1_ΔE9_ (TG) and C57BL/6J (NTG) control mice were used in this study. Mice were originally purchased from JAX MMRRC (Stock # 034829) and bred in our facility. All APP/PS1 mice were initially genotyped by PCR prior to grouping. The PCR result was further verified by measuring plasma Aβ40. Only the mice expressing Aβ40 were used as TG mice.

Individual animals were subjected to the radial arm water maze (RAWM) test before and after the treatment started. The RAWM pre-behavior results and plasma Aβ40 levels were balanced across TG groups. Prior to the start of treatment with nanoformulation, groups were balanced with respect to gender, plasma Aβ level, body weight, and baseline memory test results within gender. At the age of 12 months, the TG animals were given the intranasal treatment of the MIT nanoformulation daily for 3 months. Plasma samples prepared from whole blood were collected at 1, 1.5, and 3 months after the beginning of the treatment and stored at −80 °C. All the animals were euthanized after completing the treatment. Brain tissue samples were collected and stored in liquid nitrogen.

All procedures with animals were conducted in compliance with the National Institutes of Health guide for the care and use of laboratory animals. All animal experiments were approved by the Institutional Animal Care and Use Committee (IACUC) (Project ID: IS00000959) and performed according to the National Institutes of Health (NIH) guidelines.

THC (CAS: 1972-08-3603-001-00-X, lot SLBZ6905) and melatonin (CAS: 73-31-4) were purchased from Sigma-Aldrich (St. Luis, MO). Insulin “Degu” was purchased from Polaris Biology (Chongqing, China). Lyophilized Aβ1-42 peptide (Biomer Technology. Cat: 1409-rPEP-02) was suspended in pre-chilled 1,1,1,3,3,3-Hexafluoro-2-propanol (HFIP) on ice to make 1 mM of the Aβ solution at a concentration of 1 mM. The Aβ solution was continuously stirred at room temperature for 24 h until it completely dissolved. After that, 10 µL of the Aβ solution was aliquoted into pre-chilled tubes and centrifuged at 1000× *g* using a SpeedVac to evaporate the HFIP. The dried solutes were stored at −80 °C. Before use, Aβ was reconstituted in 1% NH_4_OH to 10 mg/mL and diluted into a working solution with 1xTris-buffered saline (TBS). All other chemicals and solvents were obtained from commercial sources.

### 2.2. MIT Nanoformulation Preparation

The oil phase, consisting of a mixture of medium-chain triglyceride oil and an emulsifying agent lecithin, was prepared in a beaker. The THC was dissolved in the oil phase containing an antioxidant, ethanol, and sonicated until the complete dissolution of the cannabinoids. In another beaker, the melatonin and insulin were dissolved in water. To obtain the MIT nanoformulation, the oil was transferred into a water phase beaker with an electrical stirrer and sonicator for about 10–15 min to make an emulsion. The pH of the emulsion was adjusted to between 6 and 7. The emulsion was passed through a homogenizer to reduce the particle size to the nanoscale. The particle size was found to be between 200 and 250 nm. This formulation contained 83 mg/mL of THC, 167 mg/mL of melatonin, and 20 U/mL of insulin. A volume of 6 µL was intranasally instilled daily so each mouse received 0.5 µg of THC, 1 µg of melatonin, and 0.2 µg of insulin. The administered dose of THC, melatonin, and insulin in the MIT nanoformulation were 0.02, 0.04, and 0.008 mg/kg, respectively.

### 2.3. Intranasal Treatment

MIT nanoformulation was administered intranasally every day for three months. The intranasal administration was conducted in accordance with the standard methods of instillation described earlier. Each mouse was gently grasped by the back of the neck with the abdomen facing upwards while 6 μL of the nanoemulsion MIT was instilled in a nostril dropwise [25].

Prior to the start of the treatment, groups were constituted according to the following criteria in order to minimize between-group variability. Groups were balanced with respect to gender, plasma Aβ levels, body weight, and baseline memory test results varied within gender.

All experiments were performed on 33 mice. For behavioral experiments, 18 middle-aged TG and 15 NTG mice were used. The NTG control mice and APP/PS1 TG mice were divided into three groups: (1) vehicle-treated NTG control mice, (2) vehicle-treated TG control mice, and (3) MIT-treatment mice.

### 2.4. Radial Arm Water Maze Behavior Study

The NTG and TG animal groups were tested for memory performance on a radial arm water maze. The RAWM is a black circular water tub with 6 V-shaped stainless-steel structures arranged to form a swimming field with an open central area and 6 arms. Mice were allowed five subsequent trials per day. Every trial started in a different start arm, which could be any of the six swim paths in the maze (except the goal arm) on a particular day for a particular mouse. The platform location was changed daily to a different arm in a semi-random pattern, and different start arms for each of the daily trials (5 trials) were selected from the remaining five swim arms in a semi-random sequence that involved all arms. On any given day of testing, four acquisition trials (Trial 1- Trial 4) and one retention trial (Trial 5) after a 30 min delay took place. For any given trial, the number of arm selection errors (number of errors) and the escape latency time (latency) prior to escape onto the platform were recorded. The tested mouse was placed in the starting arm facing the center of the pool for each trial and given 60 s to find the platform. Once on the platform, the mouse was allowed to stay there for 30 s so that it could observe visual cues before the next trial. If the animal did not find the platform within 1 min, it was guided, while in the water, toward the platform and left there for 15 s. An error was counted each time the mouse entered an arm other than the goal arm. One extra error was recorded when the mouse refused to make at least three choices during that trial. Data from the 12 days of the study were grouped into four blocks (every 3 days of data are presented as one block). The training was continued for 9 consecutive days or until the animals reached the days to criterion (DTC) in learning and in all memory tests. DTC is the number of days the animal spends to make no more than one error on three consecutive days [31]. All experiments were conducted in a dimly lit room, and the mouse had to use cues in the room to spatially memorize the location of the platform on that day.

For the evaluation of pre- and post-treatment spatial learning and memory, each mouse was tested on the RAWM device for 12 days and completed five trials per day. Nine of the twelve days were for training. Evaluation of the spatial learning and memory was based on the mean number of errors and escape latency data obtained in Trial 1 and Trial 5 during the last block.

### 2.5. Tissue Preparation and Sample Collection

Blood was collected before treatment and every 1.5 months from the submandibular vein into an EDTA-containing tube. Tubes were kept on ice and centrifuged at 300× *g* for 5 min, and collected plasma samples were stored at −80 °C.

On the day following the last behavioral assay, the animals were anesthetized with SomnaSol (Henry Schein Animal Health. Cat: 024352) and intracardially perfused with 50 mL of saline. Blood samples were taken by intracardial assay and the brain was removed with a sagittal bisection and the left half immersed in freshly prepared 4% paraformaldehyde in PBS (pH 7.4) for histopathology. The rostral portion of this half and the right half was processed for biochemical analysis. The brain tissue was immediately frozen in liquid nitrogen and stored at −80 °C.

### 2.6. Brain Tissue Protein Extraction

Frozen brain tissue was thawed and homogenized in the RIPA buffer containing proteinase inhibitor (100 mM Tris, 150 mM NaCl, 0.5% DOC, 1% nonidet P-40, 0.2% SDS, 1 mM Na_3_VO_4_, 10 mM NaF, 1 mM PMSF, 20 uM Leupeptin) with a pellet pestle motor and 10 s of sonication, then centrifuged for 20 min at 21,000× *g* at 4 °C. Crude protein concentrations were determined using the Bio-Rad DC protein assay (Bio-Rad. Cat: 5000112) and adjusted to the same level for all the samples. The supernatants obtained from this protocol were stored at −80 °C. The soluble and insoluble Aβ peptide extraction was based on the protocol by Izco M et al. with a slight modification [32]. Guanidine-HCl at a concentration of 6 M was used to dissolve the insoluble Aβ pellet.

### 2.7. Aβ1-40 and Aβ1-42 Peptide Measurement

Plasma Aβ40 and Aβ42 peptide levels and brain tissue levels of soluble and insoluble peptide levels were measured using a commercially available Aβ human enzyme-linked immunosorbent assay (ELISA) kit (MegaNano Diagnostics Inc., Tampa, FL, USA) according to the manufacturer instructions. Briefly, the brain tissues were homogenized in 400 µL of RIPA buffer and sonicated for 20 s on ice. Samples underwent centrifugation, and the supernatants were stored at −80 °C. Plasma Aβ40 and Aβ42 levels were determined from pre-treatment and during/post-treatment blood samples using the same ELISA kits. For the insoluble Aβ, the pellet after preparation for soluble Aβ was dissolved with guanidine-HCl and diluted with assay buffer to 1:5000 for Aβ measurement with the same assay as the soluble Aβ kits. Brain tissue sample Aβ40 and Aβ42 peptide concentrations are demonstrated in ng/mg of total protein. Wavelength readings were performed using a Synergy H1 Hybrid Multi-Mode Reader (BioTek Instruments, Winooski, VT, USA) and corrected by subtracting the readings at 540 nm from the readings at 450 nm. Aβ was quantified using standard curves of synthetic peptides Aβ40 and Aβ42.

### 2.8. Immunoblotting Detection for Protein Expression

Aβ, GSK3β, p-GSK3β, tau, p-tau, MFF, TFAM, CKMT1, Drp1, Fis1, Opa1, Mfn1, Mfn2, Pink1, Parkin, and β-actin expression in brain tissue were detected by an immunoblotting assay. Equal amounts of mouse brain protein samples were denatured with a loading buffer (Invitrogen. Cat: NP0007) containing 1.5% β-mercaptoethanol and heated at 75 °C for 10 min. An equal amount of the total proteins (20 µg/well) was then loaded into each well and separated using a 10% Bis-Tris gel at 160 V for 30–50 min in MES or MOPS running buffers. Prestained protein molecular weight markers were employed as molecular standards for every blot. The separated samples were transferred with a wet assay to the PVDF membrane. Membranes were first blocked with 0.2% Iblock buffer for 1 h at room temperature and then incubated with the primary antibody at designated dilutions in blocking buffer on a shaker overnight at 4 °C. After washing with PBST three times for 5 min, the blots were incubated with the appropriate horseradish peroxidase-conjugated secondary antibody in a blocking buffer for 1 h. The enhanced chemiluminescence substrate (Thermo Scientific. Cat:34078) was used to develop the blots. Image J software was used for gel quantification. The primary antibodies used for the protein detection were: Aβ R22W (MegaNano Diagnostics Inc., Tampa, FL, USA) (1:2000); p-GSK3β (Cell signaling. Cat: 9336S) (1:2000); GSK3β (Cell signaling. Cat: 9315S) (1:2000); p-tau 217 and tau (MegaNano Diagnostics Inc., Tampa, FL, USA) (1:3000); TFAM (Biolegend. Cat: 850501) (1:2000); MFF (Biolegend. Cat: 857501) (1:4000); CKMT1 (Biolegend. Cat: 867201) (1:4000); Drp1 (Cell signaling technology. Cat: 5391) (1:2000); Fis1 (ThermoFisher. Cat: PA5-22142) (1:1000); Opa1 (Proteintech. Cat: 66583) (1:2000); Mfn1 (Proteintech. Cat: 66776) (1:2000); Mfn2 (Proteintech. Cat: 67487) (1:2000); Pink1 (Proteintech. Cat: 23274) (1:2000); Parkin (Proteintech. Cat: 66674) (1:2000); and β-Actin (Sigma. Cat: A5441) (1:10000).

### 2.9. Immunohistochemistry

The left cerebral hemispheres were fixed in 4% PFA, dehydrated through a series of sucrose solutions, and sectioned at a thickness of 25 µm. The brain sections were incubated overnight at 4 °C with the primary antibodies (1:100 dilution for all) specific to the protein of interest, Aβ 9A9 (MegaNano Diagnostics Inc., Tampa, FL, USA). After incubation with the primary antibody, the brain sections were subjected to an hour’s incubation with the biotinylated secondary antibody according to the manufacturer’s protocol (Vector Laboratories). Light microscopy staining was achieved with the standard biotin-streptavidin/HRP procedure and DAB chromogen. The sections were then counterstained with hematoxylin and mounted under coverslips. For each protein of interest, three sections were selected from the same hippocampus layer of the brain and used for analysis. All three measurements were averaged for each mouse to yield the value for further statistical analysis. The abnormal, overstained, or cracks were excluded from the analysis field.

### 2.10. Data Analysis

Statistical analyses were performed using GraphPad Prism 8.0 software (GraphPad Software. San Diego, CA, USA). All results were presented as mean ± standard deviation (SD). A paired sample *t* test was used to compare two means from the same animal. Comparison of means between two independent groups was made using the two-sample *t* test. A comparison of means between more than two independent groups was made using a one-way ANOVA followed by a Tukey’s post hoc multiple comparison test. Results of latency to reach the platform and number of errors obtained from the RAWM test were analyzed using the two-way repeated measure ANOVA with time and treatment as factors.

## 3. Results

### 3.1. MIT Nanoformulation Continuous Treatment Improves APP/PS1 Aged MICE Cognitive Performance

**Figure 1:**
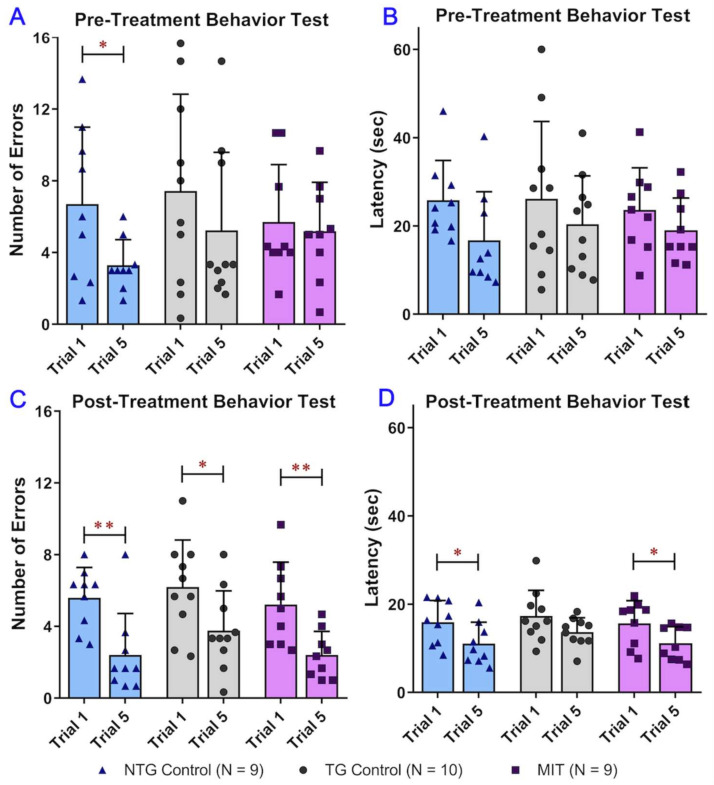
Evaluation of the spatial reference memory of 12-month-old wild-type C57BL/6 mice and transgenic APP/PS1 mice using the radial arm water maze (RAWM) test. C57BL/6 mice and transgenic APP/PS1 mice were divided into three study groups, i.e., the non-transgenic control (NTG), transgenic APP/PS1 (TG) control, and TG MIT treatment groups, and treated with vehicle control or MIT once daily for 3 months. The cognitive performances of NTG and TG mice were presented as trials of the number of errors and escape latency. Both parameters demonstrated the difference in cognitive performance at the learning phase—Trial 1—and at the memory phase—Trial 5. The values are expressed as means ± standard deviation (SD) (N = 9 for the NTG control group, N = 10 for the TG control groups, and N = 9 for the TG MIT treatment group). SD is denoted by error bars. A comparison of (**A**) baseline number of errors, (**B**) baseline latency, (**C**) the post-treatment number of errors, and (**D**) the post-treatment latency between Trial 1 and Trial 5 demonstrated a significant decrease in baseline number of errors (*p* < 0.05), post-treatment number of errors (*p* < 0.01), and latency (*p* < 0.05) in NTG control mice; a significant decrease in the post-treatment number of errors (*p* < 0.05) in TG control mice; and a significant decrease in the post-treatment number of errors (*p* < 0.01) and latency (*p* < 0.05) in MIT-treated TG mice. One-way ANOVA followed by a Tukey’s post hoc multiple comparison test showed that there was no statistically significant difference in the number of errors and latency between the three study groups. * *p* < 0.05 and ** *p* < 0.01 compared with Trial 1 using the paired-sample *t*-test.

To test the therapeutic effect of MIT nanoformulation on cognitive impairment in the progressive stage of AD, RAWM behavioral tests were conducted. For the RAWM study of working memory, 12-month-old transgenic (TG) and non-transgenic (NTG) animals were tested prior to the start of MIT treatment to establish baseline performance levels and determine if a transgenic effect was present. The RAWM study assessing an individual animal’s pre-treatment behaviors was conducted for 12 days, and the obtained data was grouped into four blocks. Every day, individual mice underwent five trials, where the very last trial was a memory test, and all previous trials were aimed at animal learning and familiarization with the RAWM device. No statistically significant differences were found in the latency and number of errors between the three study groups before the start of the treatments, suggesting the insignificance of effect modification by grouping (Figure 1A,B). A two-way ANOVA of the baseline number of errors revealed a significant effect of time (F(1, 25) = 4.270 and *p* = 0.0493), while the effects of treatment and time × treatment interaction were not significant (F(2, 25) = 0.5564 and *p* = 0.5802 for the effect of treatment; F(2, 25) = 0.6952 and *p* = 0.5084 for the effect of time × treatment interaction) (Figure 1A). A two-way ANOVA of the baseline latency data revealed a significant effect of time (F(1, 25) = 4.416 and *p* = 0.0458), while the effects of treatment and time × treatment interaction were not significant (F(2, 25) = 0.1897 and *p* = 0.8284 for the effect of treatment; F(2, 25) = 0.1812 and *p* = 0.8353 for the effect of time × treatment interaction) (Figure 1B). Results of the one-way ANOVA followed by a Tukey’s post hoc multiple comparison test indicated that there was no significant difference in latency and number of errors between the NTG control, TG control, and TG MIT treatment groups during either Trial 1 or Trial 5 (*p* > 0.05) (Figure 1A,B). These results demonstrated that the performance improved over trials but was not significantly different between groups. Results of the paired-sample *t*-test showed that NTG control mice had a significantly lower number of errors in Trial 5 than in Trial 1 (Figure 1A). No significant differences in the latency and number of errors between Trial 1 and Trial 5 were found in the TG control and MIT treatment groups. Taken together, it was demonstrated that the APP/PS1 mice displayed spatial memory deficits compared with the NTG mice before the MIT treatment was initiated. The APP/PS1 mice were grouped based on the RAWM results so that there was no overtly detectable difference in spatial memory between the three study groups.

After the 3-month treatment, results of two-way ANOVA of the number of errors demonstrated a significant effect of time (F(1, 25) = 39.03, *p* < 0.001), while no significant effects of treatment or time × treatment interaction were found (F(2, 25) = 2.239 and *p* = 0.3181 for treatment; F(2, 25) = 0.2371 and *p* = 0.7907 for time × treatment interaction) (Figure 1C). Results of a two-way ANOVA of the latency data showed a significant effect of time (F(1, 25) = 20.01, *p* < 0.001), while no significant effects of treatment and time × treatment interaction were found (F(2, 25) = 0.8800 and *p* = 0.4272 for treatment; F(2, 25) = 0.1489 and *p* = 0.8624 for time × treatment interaction) (Figure 1D). No significant differences in latency or number of errors were found between the NTG control, TG control, and TG MIT treatment groups during either Trial 1 or Trial 5 (*p* > 0.05. One-way ANOVA followed by a Tukey’s post hoc multiple comparison test) (Figure 1C,D). These observations demonstrated improved performance over trials but no significant difference in the spatial learning memory between study groups. The mean number of errors displayed by the NTG control, TG control, and MIT treatment TG mice in Trial 5 was significantly decreased by 57% (*p* < 0.01), 39% (*p* < 0.05), and 54% (*p* < 0.01), respectively, compared with the mean number of errors in Trial 1 (Figure 1C). The NTG control and MIT-treated TG mice showed a significant decline in escape latency between Trial 1 and Trial 5 (decreased by 30% and 29%, respectively. *p* < 0.05 for both.), whereas the mean escape latency displayed in Trial 5 was significantly decreased by 30% (*p* < 0.05) and 29% (*p* < 0.05), respectively, compared with the mean latency in Trial 1 (Figure 1D). Although the mean latency displayed by the TG control mice in Trial 5 was decreased by 21% compared with that in Trial 1, the difference was not statistically significant (*p* > 0.05), suggesting substantially impaired working memory in the TG control mice (Figure 1D). Overall, the superiority of the MIT-treated APP/PS1 mice over the control APP/PS1 mice was evident in that the performance of the MIT-treated APP/PS1 mice improved significantly during the RAWM test sessions, while the control APP/PS1 mice consistently exhibited inferior acquisition with relatively little improvement over the same training period.

### 3.2. Intranasal MIT Treatment Induces a Decrease in Aβ Levels in the Brain

**Figure 2:**
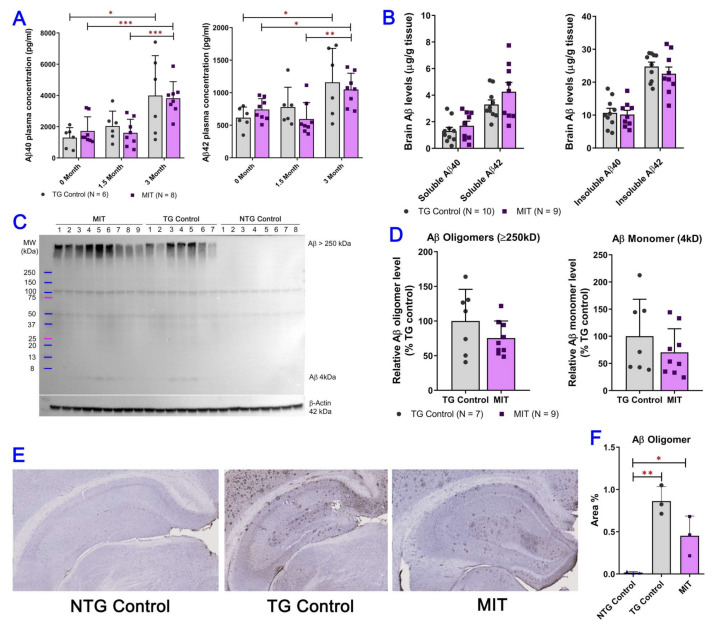
The evaluation of monomeric and oligomeric Aβ in plasma and brain tissue of APP/PS1 mice after intranasal MIT treatment for 3 months. (**A**) Aβ40 and Aβ42 levels in the plasma of APP/PS1 mice were evaluated using ELISA. Plasma samples were collected from the APP/PS1 mice at the beginning, in the middle, and after the intranasal treatment course. No significant difference in Aβ1-40 and Aβ42 plasma levels was found between the TG control (N = 6) and MIT (N = 8) treatment groups (*p* > 0.05 using an independent sample *t*-test). A comparison of plasma soluble Aβ levels measured at different time points showed that plasma levels of soluble Aβ in TG control mice measured after the 3-month vehicle treatment were significantly higher than the baseline levels. Soluble Aβ plasma levels in MIT-treated TG mice measured after the 3-month MIT intranasal treatment were significantly higher than the baseline levels and those measured after the 1.5-month treatment. * *p* < 0.05, ** *p* < 0.01, and *** *p* < 0.001 compared with measurement at 3 months after the start of the treatment using one-way ANOVA with a Tukey’s post hoc multiple comparison test. (**B**) Soluble and insoluble Aβ40 and Aβ42 peptide levels in TG control (N = 10) and MIC-treated TG (N = 9) brain tissues were evaluated using ELISA. Brain tissue samples were collected from the NTG mice treated with vehicle control and the TG mice treated with either vehicle control or MIT once daily for 3 months. (**C**) Western blotting analysis of oligomeric (MW = >250 kD) and monomeric (MW = 4 kD) Aβ in the brain tissue of MIT-treated (N = 9), TG control (N = 7), and NTG control (N = 8) mice. Neither oligomeric nor monomeric Aβ was detectable by Western blotting in NTG control brain tissues. (**D**) Densitometric analysis of aggregated and monomeric Aβ levels in the brains of aged MIT-treated TG mice and not-treated mice. Detection of β-actin was used to ensure equal sample loading per line. No significant difference in Aβ was found between the TG control and MIT treatment groups. All data are presented as mean ± SD. (**E**) Coronal slices of the left cerebral hemisphere from MIT-treated, NTG, and TG control mice. Representative Aβ oligomer staining images of brain sections. (**F**), quantification of IHC staining of Aβ. IHC results are presented as the percentage of Aβ oligomer staining positive tissue area per field in the brain. No statistically significant differences in the Aβ oligomeric isoform were found between the MIT-treated and the TG control group; however, MIT-treated mice had lower Aβ plaque loads than the TG control mice. The data are presented as mean ± SD (N = 3). Statistical analysis was conducted using a one-way ANOVA followed by a Tukey post hoc multiple comparison test.

To investigate whether prolonged daily MIT intranasal treatment could regulate Aβ levels, we quantified Aβ levels in plasma and brain tissue homogenates using the established semi-quantitative Western blotting and ELISA methods (Figure 2). Levels of Aβ in the plasma and brain tissues of the NTG control mice were not detectable by either ELISA or Western blotting (Figure 2C). Plasma levels of soluble Aβ in the TG control mice measured after the 3-month treatment were significantly higher than the baseline levels (*p* < 0.05 for both Aβ40 and Aβ42) (Figure 2A). Soluble Aβ plasma levels in the MIT-treated TG mice measured after the 3-month MIT intranasal treatment were significantly higher than the baseline levels (*p* < 0.001 for Aβ40 and *p* < 0.05 for Aβ42) and those measured after the 1.5-month treatment (*p* < 0.001 for Aβ40 and *p* < 0.01 for Aβ42) (Figure 2A). Soluble Aβ plasma levels in the MIT-treated APP/PS1 mice were not significantly different from those in the TG control mice (Figure 2A). This data suggests that the increased plasma Aβ levels are age-related. Although intranasal treatment with MIT for 3 months tended to increase soluble Aβ40 (by 30%) and Aβ42 (by 29%) levels and decrease insoluble Aβ40 (by 4.6%) and Aβ42 (by 8.9%) levels in the brain tissues of the APP/PS1 mice, there was no significant difference between the TG control and MIT-treatment groups (*p* > 0.05) (Figure 2B). Results of the Western blotting analysis of oligomeric Aβ aggregates (MW = >250 kD) and monomeric Aβ (MW = 4 kD) in the brain tissue of the TG mice demonstrated that brain concentrations of both oligomeric and monomeric Aβ in the MIT-treated APP/PS1 mice were lower than those in the brain tissue of the TG control mice, although the difference was not statistically significant (*p* > 0.05) (Figure 2C,D). Additionally, results of immunohistochemical staining showed that MIT inhibited the expression of Aβ oligomer in the APP/PS1 mouse brains (Figure 2F). Although it is generally believed that soluble Aβ reflects primarily monomeric Aβ species, while insoluble Aβ reflects aggregated forms [33], it is possible that soluble Aβ detected by ELISA contained dimeric Aβ [34].

### 3.3. Long-Term Intranasal MIT Nanoformulation Treatment Reduced Tau Phosphorylation and Increased GSK3β Phosphorylation at Ser9

**Figure 3:**
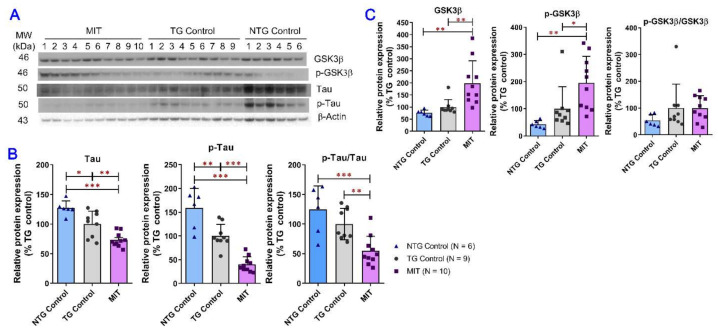
Evaluation of total and phosphorylated tau and GSK3β expression in brain tissues using the semi-quantitative Western blot analysis. (**A**) Representative immunoblots demonstrating the expression of tau, phospho-tau (p-tau), GSK3β, and phospho-GSK3β (Ser9) (p-GSK3β) proteins in brain tissues collected from individual mice. (**B**,**C**) Densitometric analysis of the relative protein expression levels of tau, p-tau, GSK3β, and p-GSK3β in the brain tissues collected from individual mice. MIT treatment significantly decreased the expression of tau and p-tau as well as the phosphorylated-to-total tau ratio compared with the vehicle control treatment in NTG (*p* < 0.001 for all) and TG (*p* < 0.01 for tau and phosphorylated-to-total tau ratio; *p* < 0.001 for p-tau) mice. MIT treatment significantly increased the expression of GSK3β and p-GSK3β compared with the vehicle control treatment in NTG (*p* < 0.01 for both) and TG (*p* < 0.01 for GSK3β; *p* < 0.05 for p-GSK3β) mice but had no significant effect on the phosphorylated-to-total GSK3β ratio. Data are presented as mean ± SD (N = 6 for the NTG control group, N = 9 for the TG control group, and N = 10 for the TG MIT treatment group). * *p* < 0.05, ** *p* < 0.01, and *** *p* < 0.001 compared between NTG control, TG control, and TG MIT treatment groups using the one-way ANOVA followed by a Tukey’s post hoc multiple comparison test.

The effect of MIT treatment on tau phosphorylation and GSK3β phosphorylation at Ser9 was examined in brain tissue homogenates to evaluate the multi-targeting potential of MIT in AD therapy (Figure 3). As shown in Figure 3B, the total tau and phosphorylated tau expression levels in the NTG control brains were significantly higher than those in the TG control brains (*p* < 0.05 and *p* < 0.01 for total and phosphorylated tau, respectively). However, when the values for phosphorylated tau were expressed as a ratio of phosphorylated to total protein, the significant difference in tau phosphorylation between the NTG and TG control groups was eliminated (Figure 3B). This result suggests that the difference in tau phosphorylation is associated with the difference in tau total abundance between the NTG and TG control mice. Intranasal treatment with MIT for 3 months significantly decreased the expression of total tau and phospho-tau as well as the phosphorylated to total tau ratio in the APP/PS1 mice compared to the NTG control (*p* < 0.001 for all) and the TG control (*p* < 0.01 for tau and phosphorylated-to-total tau ratio; *p* < 0.001 for phospho-tau) (Figure 3B). This observation suggests that MIT treatment effectively reduces tau phosphorylation in the brain.

Activation of GSK3β is associated with tau hyperphosphorylation [35,36], while phosphorylation of GSK3β at ser9 results in the inactivation of GSK3β [37,38]. As shown in Figure 3C, MIT treatment significantly increased the expression of total and phosphorylated GSK3β compared with vehicle treatment in NTG (*p* < 0.01 for both) and TG (*p* < 0.01 and *p* < 0.05 for total and phosphorylated GSK3β, respectively). The phosphorylated to total GSK3β ratio data showed no significant difference between all study groups (Figure 3C).

### 3.4. Modulation of Mitochondrial Proteins by MIT

**Figure 4:**
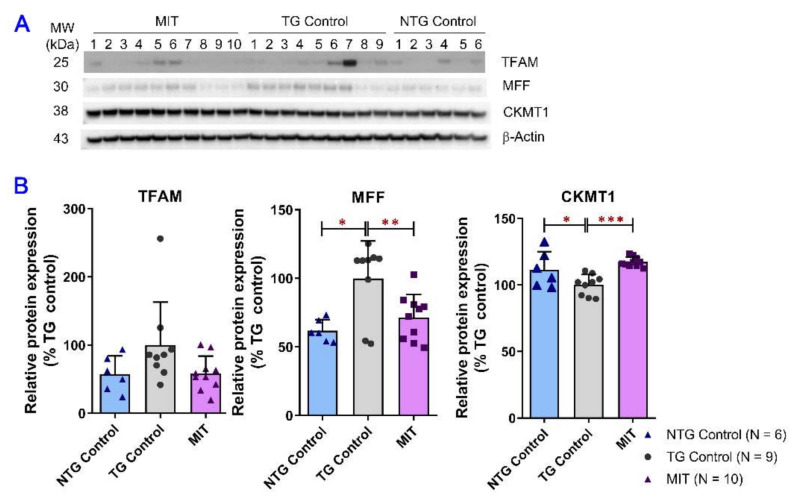
Evaluation of the MIT effect on the regulators of mitochondrial dynamics in the brain using semi-quantitative Western blot analysis. The brain tissue samples were collected from the TG and NTG control mice and TG mice treated with MIT for 3 months. (**A**) Representative immunoblots showing the expression of MFF, TFAM, and CKMT1 proteins in brain tissues collected from individual mice. (**B**) Densitometric analysis of the relative expression of MFF, TFAM, and CKMT1 proteins in brain tissues obtained from different study groups. There was a significant difference in the expression of MFF and CKMT1 proteins in NTG control (*p* < 0.05 for both) and TG MIT treatment (*p* < 0.01 and *p* < 0.001 for MFF and CKMT1, respectively) groups in comparison to the TG control group. The data are expressed as mean ± SD (N = 6 for the NTG control group, N = 9 for the TG control group, and N = 10 for the TG MIT treatment group). SD is denoted by error bars. * *p* < 0.05, ** *p* < 0.01, and *** *p* < 0.001 were compared between NTG control mice, TG control mice, and MIT-treated TG mice using a one-way ANOVA followed by a Tukey’s post hoc multiple comparison test.

Dysfunction of mitochondria correlates with neurodegenerative diseases and contributes to excessive neuronal loss in AD [39]. To assess the effect of long-term intranasal MIT treatment on AD-related mitochondrial impairments, a semi-quantitative Western blot analysis was performed to examine the protein expression levels of markers associated with mitochondrial homeostasis, bioenergetics, and fission/fusion events in the brain tissues of the NTG control, TG control, and MIT-treated TG mice. Figure 4 shows the association of AD progress with the expression of mitochondrial fission factor (MFF), creatine kinase U-type (CKMT1), and mitochondrial transcription factor A (TFAM), as well as the effect of daily MIT treatment for 3 months on the protein expression of MFF, CKMT1, and TFAM in the brain. MFF, CKMT1, and TFAM are the crucial regulators of mitochondrial fission [40], apoptosis [41], and mitochondrial homeostasis [42], respectively. As shown in Figure 4B, the mean MFF protein expression level in the brain tissues of TG control mice was significantly higher than that in the brain tissues of NTG control mice (*p* < 0.05), while MIT treatment significantly reduced the MFF expression in the brain tissues of APP/PS1 mice (*p* < 0.01). In contrast, the mean CKMT1 protein expression level was significantly lower in the brain tissues of TG control mice than that in the brain tissues of NTG control mice (*p* < 0.05), while MIT treatment significantly increased the CKMT1 protein expression in the brain tissues of APP/PS1 mice (*p* < 0.01) (Figure 4B). No significant difference in TFAM protein expression in brain tissues was detected between the NTG control, TG control, and TG MIT treatment groups (*p* > 0.05. Figure 4B). Overall, MIT treatment modulated the protein expression of MFF and CKMT1 to levels that were similar to those in the NTG control mice.

**Figure 5:**
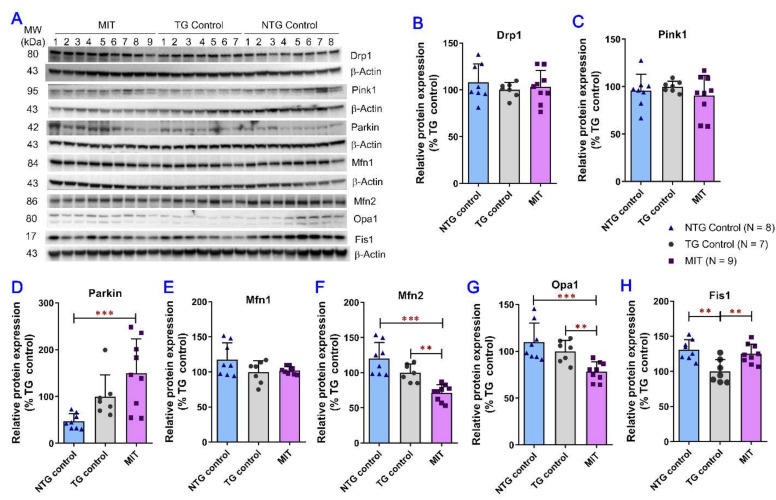
Evaluation of the MIT effect on mitochondrial fission/fusion events and mitophagy in the brain using the semi-quantitative Western blot analysis. The brain tissue samples were collected from the TG and NTG vehicle-treated control mice and the MIT-treated TG mice after the 3-month intranasal treatment. (**A**) Representative immunoblots showing the expression of Drp1, Pink1, Parkin, Mfn1, Mfn2, Opa1 and Fis1 in brain tissues collected from individual mice. Densitometric analysis of the relative expression of (**B**) Drp1, (**C**) Pink1, (**D**) Parkin, (**E**) Mfn1, (**F**) Mfn2, (**G**) Opa1, and (**H**) Fis1 proteins in brain tissues obtained from different study groups. The mean expression levels of Mfn2, Opa1, and Fis1 proteins in the brain tissues of MIT-treated APP/PS1 mice were significantly lower than those in the TG control mice (*p* < 0.01 for all). The data are expressed as mean ± SD (N = 8 for the NTG control group, N = 7 for the TG control group, and N = 9 for the TG MIT treatment group). SD is denoted by error bars. ** *p* < 0.01, and *** *p* < 0.001 were compared between the NTG control mice, TG control mice, and MIT-treated TG mice using a one-way ANOVA followed by a Tukey’s post hoc multiple comparison test.

A Western blot analysis of brain homogenate samples was conducted to further evaluate the modulatory effect of MIT treatment on the regulators of mitochondrial fission/fusion events and mitophagy. The key molecules implicated in the mitochondrial fusion process are mitofusin 1 (Mfn1), mitofusin 2 (Mfn2), and optic atrophia 1 (Opa1) [43], whereas mitochondrial fission protein 1 (Fis1) stimulates mitochondrial fission via interactions with dynamin-related protein 1 (Drp1) [44]. MIT treatment resulted in a significant decrease in the protein expression of Mfn2 and Opa1 in TG mice compared with that in the NTG control (*p* < 0.001 for both) and TG control mice (*p* < 0.01 for both) (Figure 5F,G). The mean protein expression level of Fis1 in the TG control mice was significantly lower than that in NTG control mice (*p* < 0.01) and MIT-treated TG mice (*p* < 0.01) (Figure 5H). No significant difference in Drp1 and Mfn1 protein expression was found between the NTG control, TG control, and MIT treatment groups (Figure 5B,E). With regard to the two regulators of mitophagy, PTEN-induced kinase 1 (Pink1) and ubiquitin E3 ligase (Parkin), MIT treatment resulted in a significant increase in Parkin protein expression in the TG mice compared with the vehicle treatment in NTG mice (*p* < 0.001) (Figure 5D). No significant difference in Pink1 protein expression was found between the NTG control, TG control, and MIT treatment groups (Figure 5C). Overall, it was demonstrated that MIT treatment selectively decreased Mfn2 and Opa1 protein expression and increased Fis1 and Parkin protein expression in TG mice, suggesting a modulatory effect of the MIT treatment on impaired mitochondrial fusion and fission balance and mitophagy.

## 4. Discussion

Since the novel MIT intranasal nanoformulation was developed for long-term use in AD therapy, the dose of each active ingredient in the nanoformulation was properly selected to improve cognitive functions without causing side effects. Our previous report has shown that impaired cognitive functions in aged APP/PS1 mice were restored by long-term THC intranasal treatment at 0.002 and 0.02 mg/kg [25]. Since intranasal treatment with THC at 0.002 mg/kg alleviated the AD-related memory impairment to a lesser degree than THC treatment at 0.02 mg/kg, the dose of THC used in the present study was set at 0.02 mg/kg to improve spatial learning memory without inducing any psychotropic side-effects in aged APP/PS1 mice. With regard to melatonin and insulin, melatonin is generally considered safe, while standard parenteral insulin therapy is effective in reducing blood sugar levels. Intranasal insulin administration allows the rapid and direct delivery of insulin to the brain without causing peripheral side effects [45,46]. The dose of insulin used in this study was 0.008 mg/kg, which was lower than the reported effective insulin dose of 0.64 U/kg (equivalent to 0.024 mg/kg) that resulted in a significant glucose-lowering effect in non-diabetic Balb/C mice following intravenous administration [47]. Overall, the intranasal treatment with the MIT nanoformulation is safe and suitable for long-term use in AD therapy.

The RAWM study was performed to assess the effect of MIT treatment on hippocampus-dependent spatial memory [48]. The post-treatment behavioral data indicated that the number of errors significantly decreased from Trial 1 to Trial 5 during the last trial block in all study groups (*p* < 0.01 for the NTG control and MIT treatment groups and *p* < 0.05 for the TG control group) (Figure 1C). The decrease in the number of errors in the MIT treatment group was comparable to that in the NTG control group (54% versus 57% decrease), but appeared to be greater than that in the TG control group (39% decrease) (Figure 1C). Moreover, the escape latency of the NTG control mice and MIT-treated APP/PS1 mice significantly decreased from Trial 1 to Trial 5 during the last trial block (*p* < 0.05 for both) (Figure 1D). The decrease in the escape latency of the MIT-treated APP/PS1 mice was comparable to that of NTG mice (29% versus 30% decrease) (Figure 1D). The decrease in the escape latency of the TG control mice was not significant (*p* > 0.05) (Figure 1D). Those data demonstrated that the MIT-treated mice exhibited significant improvements in spatial learning over the training period, which were comparable to the control NTG mice.

Cognitive performance impairment in aged APP/PS1 mice is associated with progressive Aβ accumulation [49]. Aβ40 is the most common isoform of Aβ in body fluids, while Aβ42 predominates in senile plaques. The amyloid plaques in Alzheimer’s brains consist of mostly Aβ42 and some plaques contain only Aβ42 [50]. Soluble Aβ is generally believed to reflect primarily monomeric Aβ species, with insoluble Aβ reflecting aggregated forms [51]. In this study, it was demonstrated that intranasal treatment with MIT in APP/PS1 mice for three months increased the mean brain concentrations of soluble Aβ40 and Aβ42 by 30% and 29%, respectively, and decreased the mean brain concentrations of insoluble Aβ40 and Aβ42 by 4.6% and 8.9%, respectively. However, the differences in the brain concentrations of soluble and insoluble Aβ40 and Aβ42 were not statistically significant between the TG control and MIT-treatment groups (*p* > 0.05) (Figure 2B). Moreover, MIT treatment reduced the mean brain concentrations of both oligomeric and monomeric Aβ in the APP/PS1 mice by 25% and 30%, respectively, while the difference between control and MIT-treated TG mice was not significant (*p* > 0.05). Immunoblotting data show that MIT induced a trend towards a decrease in oligomeric and monomeric Aβ in the brain tissue of APP/PS1 mice (Figure 2). In our previous studies, it was demonstrated that intraperitoneal administration of THC at 0.2 mg/kg every other day and intranasal administration of THC at 0.02 mg/kg once daily significantly decreased the oligomeric Aβ levels in the brain of aged APP/PS1 mice [24,25]. Moreover, oral administration of melatonin via drinking water (100 mg/L) significantly decreased mitochondrial Aβ levels in the striatum, hippocampus, and cortex of APP/PS1 mice [52]. Although an early clinical study showed that insulin promoted Aβ clearance from the brain, as reflected by the increased cerebrospinal fluid (CSF) Aβ levels in normal elderly patients [53], the effect of insulin treatment on reducing Aβ aggregation has not been documented. Taken together, our findings that the 3-month intranasal MIT treatment had the potential to reduce Aβ deposition warrant further investigation to determine whether extending the duration of MIT treatment may result in a significant inhibition of Aβ aggregation in the brain.

Abnormal hyperphosphorylation of tau is pivotally involved in the pathogenesis of AD and related tauopathies. GSK3β is a primary tau kinase that is most implicated in tau pathology in AD [54]. Although several in vitro and in vivo studies have demonstrated the inhibitory effect of melatonin on tau phosphorylation [55,56,57], melatonin was found to decrease total GSK3β expression but have no effect on phosphor- GSK3β (Ser9) expression in Neuro2A cells [58]. It was reported that insulin treatment restores tau phosphorylation to physiological levels in streptozotocin-treated C57BL/6NJcl adult mice [59], whereas the effect of insulin on rescuing tau pathology in AD models has yet to be documented. In this study, intranasal MIT treatment significantly reduced the total brain tau and phospho-tau concentrations as well as the phosphorylated-to-total tau ratio in APP/PS1 mice (Figure 3B). Similar to the findings of our previous study on intraperitoneal THC treatment in aged APP/PS mice [24], MIT treatment significantly increased brain levels of total GSK3β and phosphorylated GSK3β at Ser9, but did not have a significant effect on the phosphorylated-to-total GSK3β ratio (Figure 3C). Our data suggest that MIT induces the phosphorylation of GSK3β at Ser9 to suppress the activity of GSK3β that would otherwise be enhanced with the increased total GSK3β expression, and both GSK3β-dependent and independent mechanisms contribute to the inhibitory effect of MIT treatment on tau phosphorylation.

CKMT1, a membrane-bound mitochondrial protein that can interact with tau, is a crucial gatekeeper for mitochondrial membrane potential transition that can result in cell death through apoptosis or necrosis [41,60]. Downregulation of CKMT1 reinforces the process of programmed cell death [41]. Results of our previous study showed that intraperitoneal administration of 0.2 mg/kg of THC had no significant effect on CKMT1 expression in the brain [24]. The effect of melatonin and insulin on CKMT1 expression has not been reported. The data of this study demonstrated that MIT treatment significantly increased the protein expression of CKMT1 in APP/PS1 mouse brain tissues (*p* < 0.001. Figure 4B), suggesting that MIT treatment attenuates the toxic effect of tau that induces CKMT1-associated apoptosis.

Although the pathogenic mechanisms of AD remain incompletely understood, mitochondrial dysfunctions, including a shift in the mitochondrial fission–fusion balance towards fission, have been recognized as a prominent pathological feature of AD, as mitochondrial functions are susceptible to oxidative insults and various age-associated declines [61,62]. In this study, MIT treatment significantly counteracted the increased MFF expression (*p* < 0.01. Figure 4B) and decreased Fis1 expression (*p* < 0.01. Figure 5H) but had no effect on Drp1 protein expression (Figure 5B). Since MFF and Fis1 act independently to recruit Drp1 to ER-mitochondria contact sites and eventually induce mitochondrial fission [63], the opposite effect of MIT on MFF and Fis1 protein expression resulted in no significant change in Drp1 expression in the APP/PS1 mouse brain (Figure 5B), suggesting MIT treatment has little effect on mitochondrial fission. However, MIT treatment resulted in a significant decrease in the expression of two mitochondrial fusion-related proteins, Mfn2 and Opa1, in TG mice compared with that in the NTG control (*p* < 0.001 for both) and TG control mice (*p* < 0.01 for both) (Figure 5F, G), implicating that MIT treatment potentially reduces mitochondrial fusion. Although the reduced mitochondrial fusion represents impaired mitochondrial function [62], it may promote the removal of dysfunctional mitochondria by mitophagy [64]. This speculation is confirmed by our data showing that MIT treatment significantly increased the protein expression of Parkin (*p* < 0.001 compared with NTG control. Figure 5D), which can trigger multiple mechanisms of mitochondrial removal and regeneration [65]. Overall, our data suggest that MIT treatment has little effect on mitochondrial fission, but decreases mitochondrial fusion, which results in a compensatory increase in the removal of dysfunctional mitochondria by mitophagy and the stimulation of mitochondrial biogenesis.

## 5. Conclusions

The results of the present study provide the first evidence that MIT nanoformulation containing melatonin, insulin, and THC has potential as a multi-targeting treatment for AD. The memory-improving effect of MIT intranasal treatment is associated with its remarkable inhibitory effect on tau phosphorylation in the brain and with its modulatory effect on mitochondrial fission and fusion dynamics that lead to the compensatory enhancement of mitophagy and stimulation of mitochondrial regeneration.

## Data Availability

The data presented in this study are available on request from the corresponding authors.
